# Editorial: Recent Advances in Thermally Activated Delayed Fluorescence Materials

**DOI:** 10.3389/fchem.2020.625910

**Published:** 2020-11-30

**Authors:** Chihaya Adachi, Guohua Xie, Sebastian Reineke, Eli Zysman-Colman

**Affiliations:** ^1^Center for Organic Photonics and Electronics Research (OPERA), Kyushu University, Fukuoka, Japan; ^2^Hubei Key Laboratory on Organic and Polymeric Optoelectronic Materials, Department of Chemistry, Sauvage Center for Molecular Sciences, Wuhan University, Wuhan, China; ^3^Dresden Integrated Center for Applied Physics and Photonic Materials (IAPP), School of Science, Technische Universität Dresden, Dresden, Germany; ^4^Organic Semiconductor Centre, EaStCHEM School of Chemistry, University of St Andrews, St. Andrews, United Kingdom

**Keywords:** thermally activated delay fluorescence (TADF), OLED - organic light emitting devices/display, organic semiconductor (OSC), metal complexes, polymers

Thermally activated delayed fluorescence (TADF) materials design has rapidly evolved over the past 8 years since Adachi and co-workers demonstrated that organic donor-acceptor compounds could recruit both singlet and triplet excitons in electroluminescent devices, reaching nearly 100% internal quantum efficiency (Uoyama et al., 2012). Accompanying new and improved TADF materials has been the enhanced performance of the organic light-emitting diodes (OLEDs), underpinned by a refined understanding of the TADF mechanism and the design rules that govern materials design.

This themed issue of Frontiers in Chemistry documents recent advances in TADF materials. Paisley et al. reviewed stimuli-responsive TADF in polymer nanoparticles and how these are relevant to sensing and imaging. Yin et al. provided an overview of TADF polymer materials. Etherington summarized the solid-state solvation effects and aggregate effects in organic TADF emitters. Barman et al. reviewed mechanochromic organic TADF materials. Lee et al. focused on boron containing TADF materials. To et al. contributed a perspective on metal complexes that showed TADF and their use as emitters in OLEDs. Hamze et al. demonstrated highly efficient deep blue luminescence of 2-coordinate coinage metal complexes. Crucho et al. reported on TADF dye-loaded nanoparticles for fluorescence live-cell imaging. Li Y. et al. showed how through-space charge transfer states can be recruited within polymers to produce high-performance solution-processed OLEDs, while Chen et al. showed how similar excited states can be exploited in small molecule TADF emitters. Mubarok et al. likewise exploited through-space CT states in triarylborane-containing TADF emitters. Kusakabe et al. revealed the importance of the relative orientation of donor and acceptor groups in through space TADF small molecule emitters. TADF polymer design was featured in the work of Yang et al. who reported white OLEDs while red OLEDs were reported by Zhan et al.. Franco et al. demonstrated how TADF absorption can be sensitized by using variable length oligo(phenylene ethynylene) antennae. Cai et al. revealed how aggregation-induced delayed fluorescence materials can be recruited for high-performance solution-processed OLEDs. Li H. et al. provided examples of small molecule emitters that showed aggregation-induced delayed fluorescence. Distinct high triplet energy ambipolar TADF host materials were independently reported by Rodella et al. for vacuum-deposited OLEDs and Godumala et al. for solution-processed OLEDs. High-performance OLEDs also require horizontally oriented emitters. Naqvi et al. revealed molecular design rules for small molecular TADF emitters to exhibit more preferentially horizontal orientation. Sasabe et al. demonstrated how molecular orientation could be controlled in carbazole-containing host materials. Improved performances of blue TADF OLEDs have been of particular focus within the OLED community. Zhang et al. reported on a family of blue emitters containing an OBO-Fused Benzo[fg]tetracene acceptor. Sohn et al. demonstrated high-performance blue OLEDs using a pyrimidine-containing TADF emitter. Tsuchiya et al. showed how computational modeling can effectively guide blue emitter design. The greater exploration of chemical space has led to unorthodox emitter design. For instance, delayed fluorescence was reported by Pandey et al. in compounds containing dianthrylboron-based donor–acceptor systems, while Krotkus et al. showed fast delayed fluorescence in pyridazine-based emitters.

The articles in this themed issue provide insight into how TADF materials design has evolved and how the use of TADF materials has expanded beyond OLEDs to sensing and imaging. Despite the intense focus over the past 8 year, this collection clearly demonstrates that there is still much to learn and much from which to be inspired.

## Author Contributions

All authors listed have made a substantial, direct and intellectual contribution to the work, and approved it for publication.

## Conflict of Interest

The authors declare that the research was conducted in the absence of any commercial or financial relationships that could be construed as a potential conflict of interest.
